# Sustainable science mapping: benchmarking green AI against transformers for cross-disciplinary abstract classification using arXiv

**DOI:** 10.1038/s41598-026-48795-7

**Published:** 2026-05-15

**Authors:** Mehmet Ali Erkan, Ceylan Yozgatlıgil

**Affiliations:** Department of Statistics, Middle East Technical University, Ankara, 06800 Turkey

**Keywords:** Automated subject indexing, Academic text classification, Green AI, Deep learning in bibliometric, Computational efficiency, Engineering, Mathematics and computing

## Abstract

The exponential growth of scholarly literature necessitates automated, scalable systems for organizing knowledge domains. However, text classification of academic abstracts presents distinct challenges due to specialized terminology and diverse discourse structures across disciplines. This study proposes a resource efficient deep learning methodology to categorize academic abstracts, scaling from coarse grained domains (arXiv) to fine grained disciplinary hierarchies (Web of Science). Systematic comparative analysis of Recurrent Neural Networks (Attention-GRU) and Transformer based architectures (BERT, SciBERT) are conducted, specifically focusing on the trade-off between predictive accuracy and computational efficiency. Extensive experiments on massive benchmarks that include the WOS-46985 dataset with 134 sub-disciplines, reveal a notable finding: Our proposed Attention-based GRU model utilizing static GloVe embeddings achieved a Macro-F1 score of 0.920, achieving higher performance than leading domain specific models such as SciBERT (F1: 0.867). Furthermore, this accuracy was achieved with over 3$\times$ faster training times and significantly lower estimated energy proxy compared to Transformer variants. This research contributes to the field by providing a systematic evaluation of “Green AI” architectures, demonstrating that computationally efficient models can robustly handle the linguistic diversity of high cardinality, fine grained scientific taxonomies without the prohibitive estimated energy costs of Large Language Models.

## Introduction

The management of the exponentially growing volume of scholarly literature has become one of the most pressing challenges in information science and bibliometrics. As scientific production accelerates, traditional manual methods of subject indexing and categorization are rendered insufficient, necessitating automated systems capable of organizing vast knowledge domains efficiently. In this context, text classification serves as a cornerstone for modern information retrieval, enabling researchers to navigate complex disciplinary landscapes ranging from literature reviews to large-scale science mapping^1,2^.

Recent advances in Deep Learning techniques have revolutionized text classification, with transformer based architectures achieving high performance in different Natural Language Processing tasks^3,4^. However, the application of these powerful models to the academic domain remains fraught with challenges. Existing studies predominantly rely on general purpose benchmarks (e.g., newswire, sentiment analysis) or single domain evaluations, which fail to capture the nuanced terminologies and discourse structures inherent to heterogeneous academic disciplines. More critically, the pursuit of predictive accuracy has often overshadowed the rising computational costs associated with complex architectures. While “Green AI” has emerged as a vital paradigm advocating for resource-conscious modeling^5,6^, systematic evaluations that balance classification accuracy with estimated energy proxy and training efficiency in the context of bibliometric mining are largely absent from the literature.

**Fig. 1:**
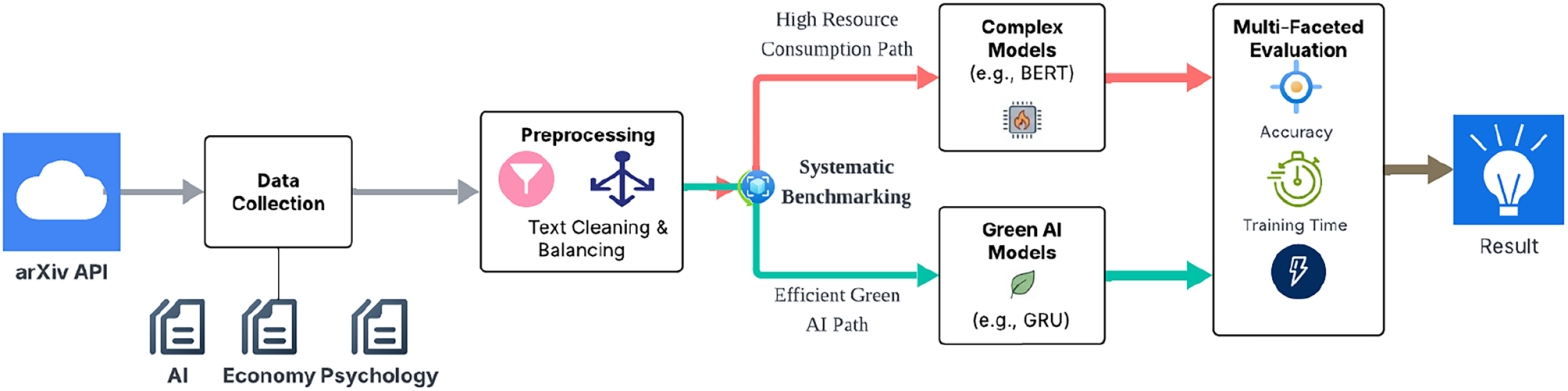
Main Flowchart.

This article addresses these gaps by proposing a resource efficient framework for cross-domain classification. A balanced arXiv dataset (AI, Economics, Psychology) is initially utilized as a controlled testbed to explore interdisciplinary overlaps. Crucially, addressing the need for fine grained validation, the evaluation is extended to the massive Web of Science (WOS) benchmarks, specifically the WOS46985 dataset, in which abstracts are classified into 134 distinct sub-disciplines. This setup allows us to test whether lightweight models can handle high-cardinality, fine-grained taxonomies where semantic distinctions are subtle. The main workflow of the framework is illustrated in Fig. 1.

Through this two tiered methodology (arXiv for exploration, WOS for scalability), the paper aims to benchmark Recurrent Neural Networks (LSTM, GRU) against Transformer based models (BERT variants). Unlike previous studies, our paper presents a comprehensive assessment that considers predictive performance, computational efficiency, and domain generalization^7^.

By bridging the gap between advanced NLP techniques and sustainable bibliometric practices, this work makes three key contributions:

1. It provides a systematic benchmark for academic abstract classification, scaling from coarse-grained domains (3 classes) to fine-grained disciplinary hierarchies (134 classes) which demonstrate the model’s robustness in real world science mapping.
2. It delivers a comprehensive efficiency performance trade-off analysis showing within the evaluated benchmark settings, Attention-based GRU models with static embeddings can outperform heavy Transformers (e.g., SciBERT) in accuracy while offering over $\sim 3\times$ speedup, providing empirical support for the Green AI paradigm.
3. It offers empirical evidence on “Semantic Stability” in scientific text which suggests that the static nature of academic terminology can render dynamic attention mechanisms computationally redundant for specific classification tasks.

To guide this investigation, we address the following research questions:

**RQ1:** How do resource-efficient architectures compare with computationally intensive Transformers in classifying both broad and fine-grained academic abstracts?

**RQ2:** What are the quantifiable trade offs between predictive performance and computational costs (latency, estimated energy, memory) across these architectures?

**RQ3:** Can a streamlined “Green AI” model (Attention-GRU) surpass domain specific Transformers (e.g., SciBERT) on complex taxonomies, offering a sustainable alternative for large scale bibliometrics?

The remainder of this paper is structured as follows: Section II reviews the evolution of deep learning in text classification. Section III details the data and methodology. Section IV presents the experimental results on both arXiv and WOS datasets. Finally, Section V discusses the implications for sustainable science mapping.

## Related work

The accelerating expansion of scientific literature has positioned automated text classification as a cornerstone of modern bibliometrics and information retrieval^8^. As manual indexing becomes increasingly infeasible, the development of robust, scalable classification systems is essential for tasks ranging from knowledge discovery to automated content analysis. This section reviews the evolution of these systems, focusing specifically on the trade-offs between predictive performance and computational sustainability in the context of academic text processing.

The transition from traditional machine learning to deep learning has revolutionized how scholarly content is processed. While Transformer-based models, particularly BERT and its domain-specific variants (e.g., SciBERT), have demonstrated high performance across various NLP tasks^9^, their application to large-scale bibliometric mining is not without challenges. Recent surveys indicate that while these large language models (LLMs) excel in capturing semantic nuances, their substantial computational demands often hinder practical deployment in real time academic information systems^10^. Consequently, there is growing interest in scalable, ML-driven frameworks capable of real time trend forecasting in large scholarly corpora, as demonstrated by recent systems like BiBLoX^11^. Conversely, RNNs offer a resource-efficient alternative, yet comparative studies benchmarking them on fine-grained academic tasks remain limited^12,13^.

A critical yet often overlooked aspect of model selection is computational efficiency, a core tenet of the emerging “Green AI” paradigm^14^. For bibliometric applications dealing with millions of records, training time and estimated energy proxy metrics are as critical as accuracy^15^. A pivotal timing analysis revealed that lighter architectures such as CNNs and RNNs can be trained significantly faster than Transformer encoders. The experiments demonstrated a consistent hierarchy in computational cost: $\text {CNN}< \text {RNN}< \text {GRU}< \text {LSTM} \ll \text {Transformer} < \text {BERT}$^5^. This suggests that for many bibliometric tasks, simpler neural architectures or even traditional machine learning models can rival complex Pretrained Language Models (PLMs) in performance while requiring a fraction of the resources^16^. Recent studies further emphasize that the high adaptation costs of specialized transformers may limit their broad accessibility for institutional repositories^17^, underscoring the need for energy aware model design^18,19^.

In addition, the effectiveness of static word representation models such as GloVe and Word2Vec in scientific text classification should be re-evaluated from the perspective of Green AI. Unlike other domains where text is used, in scientific text, semantic stability is high. Recent advancements in related NLP tasks have highlighted the robust efficacy of combining optimized word embeddings with recurrent neural networks. For instance, the integration of domain-trained embeddings with Bi-LSTM architectures has been shown to effectively capture implicit semantic regularities and nuanced text features without the need for massive Transformer pre-training or extensive handcrafted rules^20^. Previous bibliometric studies suggest that for corpora where technical jargon possesses fixed definitions, static embeddings can effectively capture semantic relationships with significantly lower dimensionality than dynamic encoders^21^. This implies that if the semantic shift of terms is minimal within a discipline, the heavy computational overhead of attention mechanisms may be redundant^22^.

The interdisciplinary nature of modern science necessitates classification models capable of generalizing across diverse semantic fields. Significant improvements in cross-domain classification can be achieved using multi-task learning models, such as the SciBERT-HSLN framework^23^. Similarly, pre-trained representations could reduce feature divergence between source and target domains, facilitating better transfer learning in specialized tasks like information security^24^. However, the extent to which these transfer capabilities hold for distinct yet overlapping fields like AI, Economics, and Psychology, where vocabulary divergence can be subtle, remains an open question, especially given the rise of ’Computational Social Science’^25^. Recent benchmarks on large scale scientific corpora specifically highlight the trade-offs between domain specific pre-training and computational overhead. For instance, a comprehensive comparative study fine-tuning LLMs like BERT, SciBERT, BioBERT, and BlueBERT on Web of Science datasets (WOS-46985, WOS-11967) demonstrated that domain specific models, particularly SciBERT, consistently outperformed general purpose models like BERT in both abstract and keyword based classification tasks, achieving high F1-scores due to their specialized vocabulary adaptation^26^. However, this performance comes at a significant computational cost. Addressing this efficiency gap, Nazarov and Tolcheev^27^ explored less resource-intensive alternatives on the same WOS-46985 dataset. While they confirmed BERT’s leadership in raw accuracy (F1: 0.908 on simple samples), they revealed that lighter architectures, such as a combination of FastText and Convolutional Neural Networks (CNN), could achieve competitive results (F1: 0.888) with drastically reduced training times and memory requirements. They emphasized that for complex, hierarchical samples (134 classes), the performance gap between heavy Transformers and optimized lightweight models narrows, making the latter a viable compromise for resource constrained environments^27^. These findings collectively underscore the need for ’Green AI’ frameworks that can leverage the semantic stability of scientific text without the excessive carbon footprint of massive Transformer ensembles.

The adoption of machine learning methodologies in social sciences has led to a convergence in vocabulary (e.g., ’optimization agents’ in both Economics and AI), creating a lexical overlap that poses distinct challenges for automated classifiers^28^. This study aims to bridge these gaps by evaluating whether computationally efficient models can robustly handle such interdisciplinary classification challenges without the environmental and resource costs of large scale Transformers.

## Methodology

This study employs a systematic workflow to evaluate the trade-off between predictive accuracy and computational efficiency. The methodology is divided into three stages: (i) data acquisition (arXiv and Web of Science), (ii) preprocessing, and (iii) deep neural network modeling which focus on the comparison between Green AI architectures and Pre-trained Language Models.

### Data collection and corpus characteristics

To construct a representative dataset of interdisciplinary scholarship, the arXiv API and the xml.etree.ElementTree library ^29^ were utilized to harvest academic abstracts from three distinct domains: Artificial Intelligence (AI), Economics (ECON), and Psychology (PSY). To assess model robustness across different data scales, two balanced datasets were created: a smaller corpus of 1,200 abstracts (400 per class) and a larger corpus of 3,000 abstracts (1,000 per class). Ground truth labels were derived directly from arXiv metadata categories.

Statistical analysis revealed distinct characteristics across domains. AI abstracts are generally longer (Mean: 156.3 words, SD: 42.7) compared to Economics (Mean: 142.8 words, SD: 38.2) and Psychology (Mean: 148.9 words, SD: 40.1). Furthermore, cross-domain vocabulary analysis indicates a 67% terminological overlap between AI and Economics, highlighting the challenge of disambiguating these fields.

**Fig. 2:**
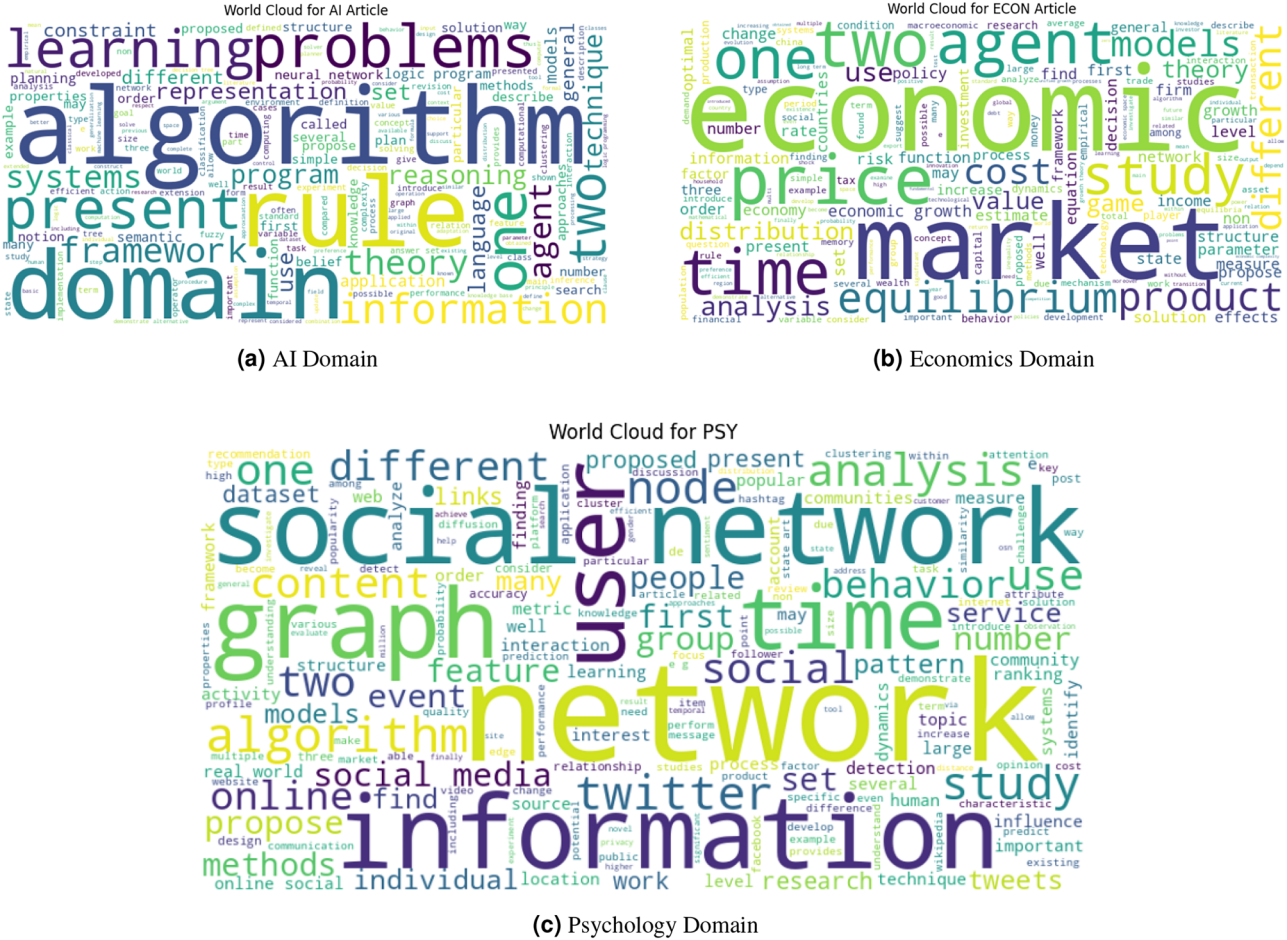
Word cloud visualizations revealing dominant themes: (**a**) AI focuses on algorithms and learning; (**b**) Economics highlights market dynamics and equilibrium; (**c**) Psychology centers on social networks and behavior.

As illustrated in Fig. 2, despite the vocabulary overlap, distinct thematic clusters exist. AI articles blend rule based systems with machine learning, Economics emphasizes theoretical models like game theory and Psychology explores behavioral patterns in digital contexts.

#### External validation: web of science (WOS) datasets

Two benchmark datasets from the Web of Science were incorporated to address the need for fine grained validation and scale^30^:

- **WOS-11967:** Contains 11,967 abstracts divided into 35 sub-disciplines (Level-2 categories), organized under 7 parent domains.
- **WOS-46985:** A massive dataset of 46,985 abstracts classified into 134 fine-grained sub-disciplines. This dataset serves as the ultimate stress test for model robustness against high-cardinality and semantic ambiguity.

### Preprocessing pipeline and feature engineering

Raw abstracts were carefully preprocessed to ensure uniform text structure and to reduce noise. The pipeline has these steps:

- **Label Encoding:** Categorical labels (“AI”, “ECON”, “PSY”) were mapped to numerical values (0, 1, 2). The dataset was subsequently split into training (70%), validation (15%), and test sets (15%) using stratified sampling to maintain class distribution.
- **Text Normalization:** Lowercase conversion, punctuation removal via regular expressions, and a custom domain specific cleaning process including mathematical notation standardization, citation normalization, and acronym expansion were applied.
- **Stopword Removal:** A custom list of simplified stopwords was filtered out to retain domain-specific terminology while reducing feature space dimensionality.

#### Sequence length and tokenization strategies

Sequence length standardization is critical for batch processing in neural networks. Statistical analysis of our corpus informed the padding strategy:

- 95th percentile length: 312 tokens
- Mean length: 187.4 tokens
- Selected Optimal Length: 256 tokens (minimizing information loss while optimizing memory).

Whitespace and punctuation based tokenization was applied for RNN-based models (LSTM, GRU). Words that were not in the vocabulary were replaced with “unknown.” The specific WordPiece tokenizer was used to handle sub-word structures well for Transformer models like BERT.

### Neural network architectures

Two families of deep learning models are benchmarked: Recurrent Neural Networks (RNNs) optimized for efficiency, and Transformer based models optimized for semantic capability.

#### Bi-LSTM text classification model

To capture sequential dependencies in both forward and backward directions, Bidirectional LSTM (Bi-LSTM) was implemented.

Let us define a sequence of input tokens as $X = (x_1, x_2, \ldots , x_L)$ where *L* represents the sequence length and each $x_t \in \{1, 2, \ldots , V\}$ is a discrete token from a vocabulary of size *V*. Each token is mapped to a continuous representation through an embedding matrix $E \in \mathbb {R}^{V \times D_{\textrm{emb}}}$, where $D_{\textrm{emb}}$ denotes the embedding dimension.

The forward hidden states $\overrightarrow{h}_t \in \mathbb {R}^{D_{\textrm{hid}}}$ are computed through the standard LSTM recurrence relation. The LSTM cell comprises three gating mechanisms, input ($i_t$), forget ($f_t$), and output ($o_t$), along with a candidate activation $g_t$.

**Fig. 3:**
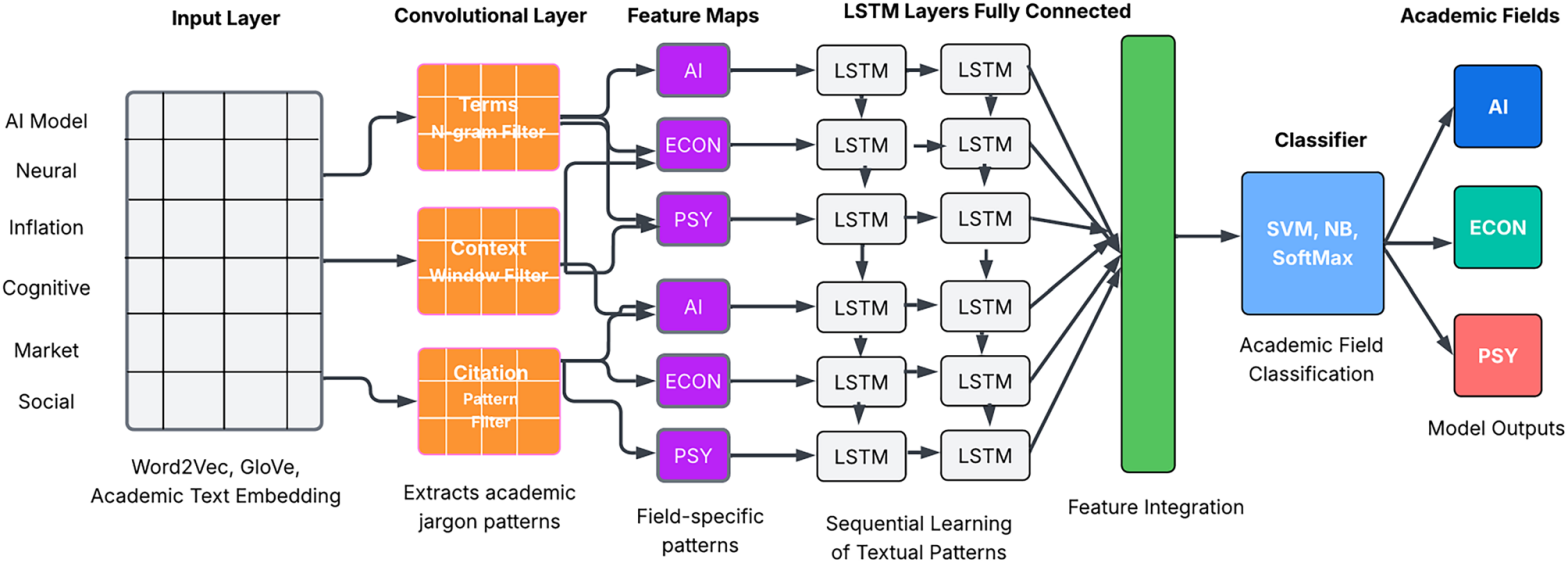
Architecture of Bidirectional LSTM with forward and backward processing streams. The final hidden states from both directions are concatenated to form the sequence-level representation for classification.

Then, based on the Fig. 3, the bidirectional mechanism captures both left and right context. The final sequence representation $h^*\in \mathbb {R}^{2D_{\textrm{hid}}}$ is formed by concatenating the final forward and backward hidden states.

#### Proposed green AI model: attention-based GRU

To maximize computational efficiency while addressing the semantic complexity of fine-grained bibliometric classification (e.g., 134 sub-disciplines), an optimized Attention-based Gated Recurrent Unit (Bi-GRU) architecture is proposed. While standard RNNs often struggle with information bottlenecks in long sequences, our proposed framework integrates a specific attention layer to selectively weigh the importance of key terminologies within abstracts.

**Fig. 4:**
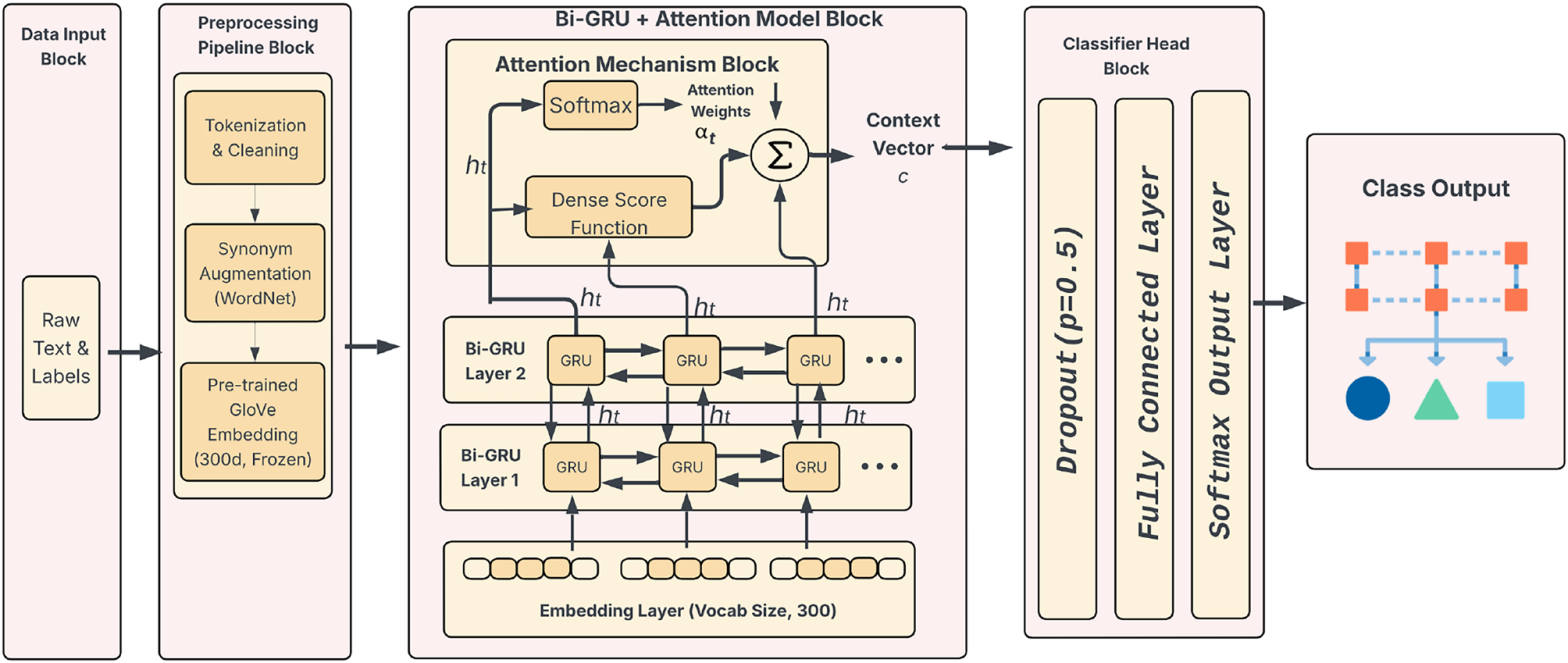
Schematic architecture of the proposed Attention-based Bi-GRU model. The design emphasizes inference efficiency by utilizing frozen GloVe embeddings and a streamlined Attention Mechanism. The raw abstract is processed through bidirectional recurrent layers, weighted by attention scores to form a context vector *c*, which is then mapped directly to class probabilities. This lightweight structure avoids the computational complexity of Transformer encoders.

As illustrated in Fig. 4, the core of the model utilizes the Gated Recurrent Unit (GRU), which offers a streamlined alternative to LSTM by merging the forget and input gates into a single *update gate* ($z_t$). This architectural reduction decreases the parameter count by approximately 25% compared to LSTM ($3(D^2+D_{emb}D)$ parameters vs. $4(D^2+D_{emb}D)$), directly associate with “Green AI” principles by reducing floating-point operations (FLOPs) during training. Unlike standard pooling strategies (e.g., using only the final hidden state), a soft attention mechanism was implemented. Let $H = [h_1, h_2, \dots , h_L]$ represent the sequence of hidden states from the Bi-GRU layer. The attention mechanism computes a context vector *c* as a weighted sum of these states:

1 $$\begin{aligned} \alpha _t = \text {softmax}(w^T \tanh (W_h h_t + b_h)), \quad c = \sum _{t=1}^{L} \alpha _t h_t \end{aligned}$$

This allows the model to dynamically focus on keywords that help distinguish between ’market equilibrium’ in Economics and ’Nash equilibrium’ in AI. This mitigates the ’vanishing context’ problem in long scientific abstracts. Frozen GloVe 300d embeddings are used instead of training dynamic embeddings from scratch, further reducing the estimated computational energy proxy. Our hypothesis, corroborated by the findings, is that scientific terminology demonstrates significant “semantic stability,” rendering high-dimensional static vectors adequate for encapsulating domain nuances without the exorbitant expense of fine-tuning extensive Transformer embedding layers.

### Methodological note on energy estimation

Energy consumption was not directly measured via hardware telemetry or monitoring utilities. An estimated energy proxy is computed using the formula: $E_{proxy} \approx P_{TDP} \times t_{training}$, where $P_{TDP}$ is the Thermal Design Power of the hardware (assumed at 250W for the NVIDIA V100 PCIe) and $t_{training}$ is the wall-clock training duration. This provides an upper bound estimate of the energy consumed by each neural architecture during training. Some assumptions have to be made to obtain this number: (i) constant hardware utilization at the rated TDP throughout the entire training phase; (ii) no power-capping mechanisms applied; (iii) the exclusion of CPU, memory, and storage power overheads and (iv) idle power consumption is not subtracted. This proxy approach standardizes the reporting metric, providing a reliable baseline for comparing the relative environmental impact of different neural architectures regardless of specific local deployment infrastructure.

## Experimental implementation

To ensure reproducibility benchmarking, this section details the specific architectural configurations, hyperparameter settings, and training protocols employed for each model family. All experiments were conducted using the PyTorch framework on an NVIDIA V100 GPU.

### Reproducibility and hyperparameter configuration

To ensure complete transparency and reproducibility, all experiments were conducted under strictly controlled settings. To capture initialization variance while maintaining reproducibility across the 15 independent runs, a sequential range of fixed random seeds (e.g., 42 through 56) was utilized. All models were trained and evaluated on a single NVIDIA GPU.

To account for the linguistic structure of the datasets, the vocabulary was constrained to the 15,000 most frequent tokens, and sequences were padded or truncated to a maximum length of 250 tokens. Furthermore, to mitigate the effects of class imbalance during training, dynamic class weights were calculated and integrated directly into the Cross-Entropy loss function.

Table 1 summarizes the consolidated hyperparameter configurations. For the optimization of our proposed Attention GRU model, the Adam optimizer was utilized with L2 regularization (weight decay = $1 \times 10^{-4}$). Instead of a static learning rate, a ReduceLROnPlateau scheduler was implemented, which dynamically reduced the learning rate by a factor of 0.5 if the validation accuracy did not improve for 2 consecutive epochs. To systematically determine the most effective architectural settings, we used Weights & Biases (W&B) to conduct a Bayesian hyperparameter sweep. As illustrated in Table 8, this tuning process executed multiple runs to identify the global optima, with the best performing configuration (Run ID: rose-sweep-8) achieving a validation accuracy of 96.11% and directly informing our final parameter selection.

**Table 1:** Consolidated Hyperparameter Configuration for Evaluated Models.

Model	Layers	Hidden Dim.	Dropout	Learning Rate	Batch Size	Max Epochs
LSTM	2	128	0.5	$1 \times 10^{-3}$	32	20
LSTM (GloVe)	3 (Bi)	64	0.0	$6.2 \times 10^{-4}$	32	20
**Attention-GRU**	**2 (Bi)**	**256**	**0.5**	$\mathbf {8 \times 10^{-4}}$	**64**	**20**
BERT (Base)	12	768	0.1	$2 \times 10^{-5}$	32	5
DeBERTa	12	768	0.1	$2 \times 10^{-5}$	32	5
SciBERT	12	768	0.1	$2 \times 10^{-5}$	32	5

#### Bi-LSTM configurations

**Fig. 5:**
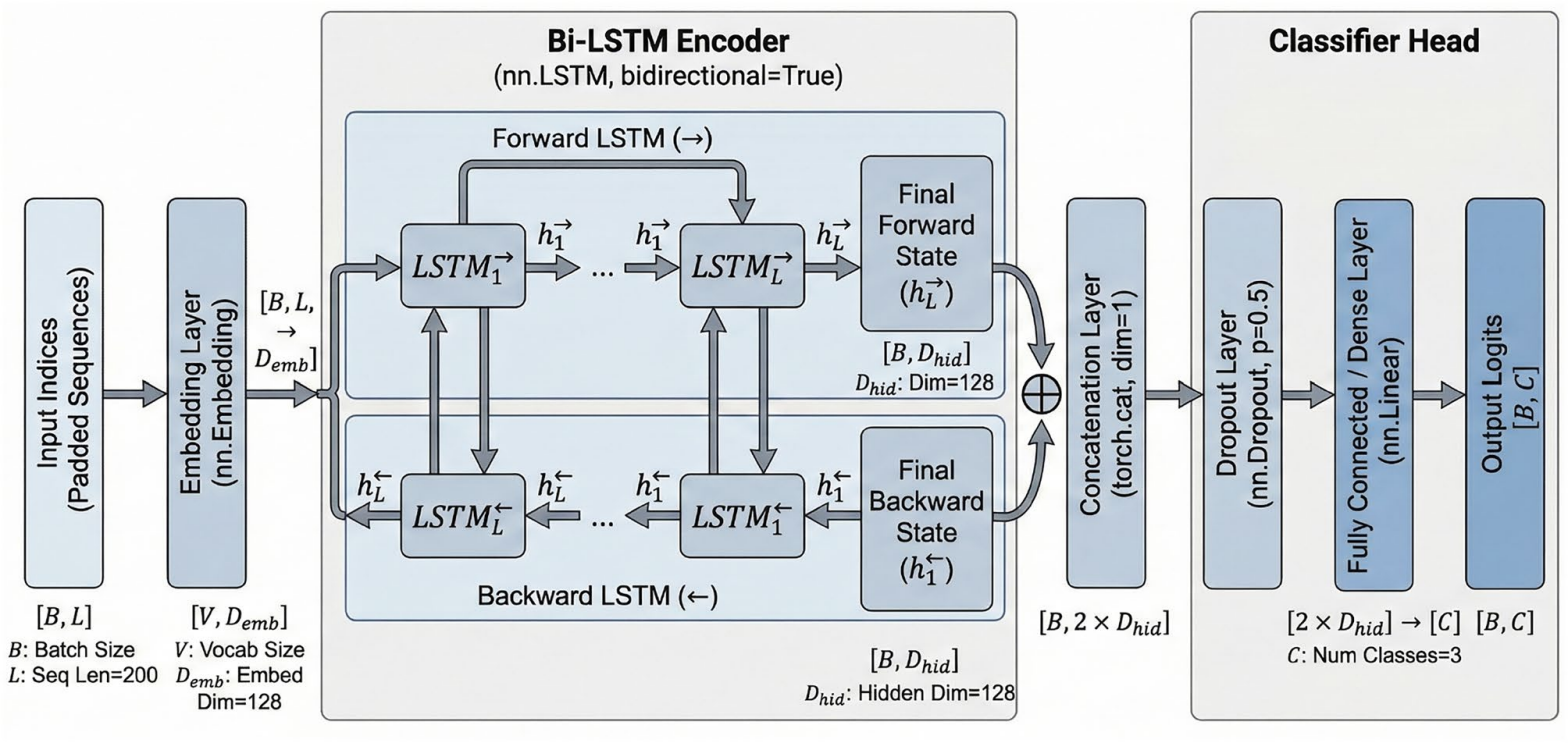
Detailed architecture of the Bidirectional LSTM text classification model. Tensor shapes are indicated in brackets [ ]. Key parameters used in this configuration: $L=200$ (Sequence Length), $D_{emb}=128$ (Embedding Dimension), $D_{hid}=128$ (Hidden Dimension), $C=3$ (Number of Classes).

**Fig. 6:**
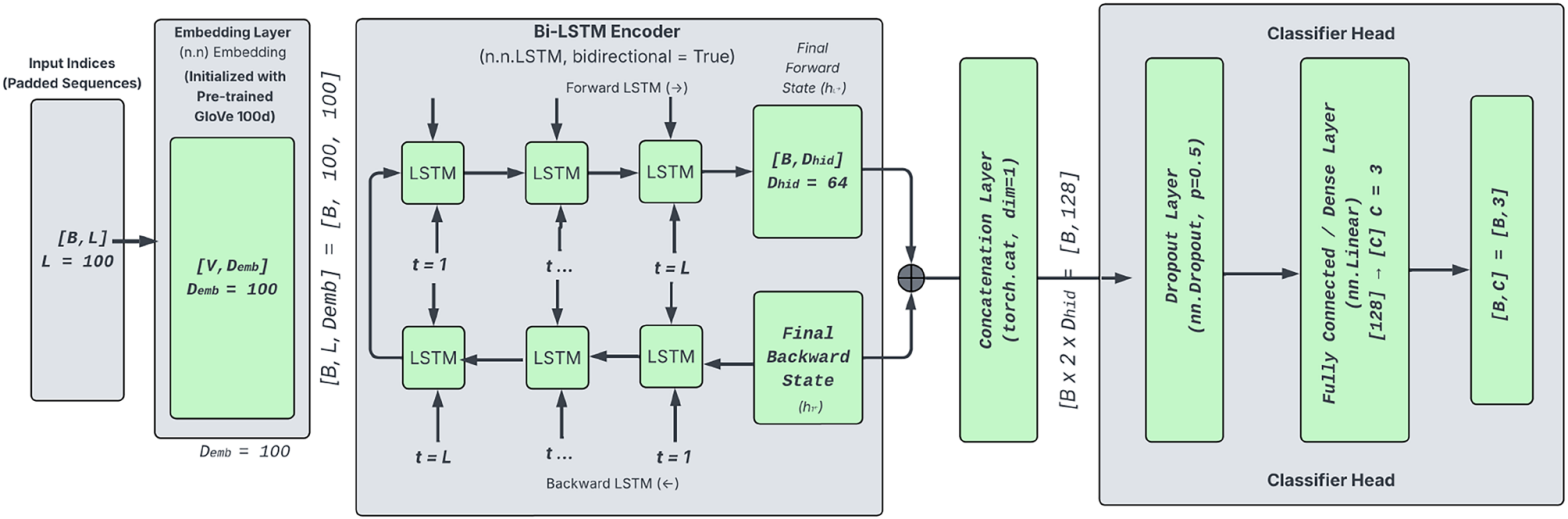
Architecture of the Bi-LSTM model utilizing pre-trained GloVe embeddings. The diagram highlights the initialization of the embedding layer with 100-dimensional GloVe vectors. Specific hyperparameters derived from the code: $L=100$ (Seq Len), $D_{emb}=100$ (GloVe Dim), $D_{hid}=64$ (Hidden Dim), $C=3$ (Classes).

In the LSTM section, two distinct models(Figs. 5 and 6) were implemented with comprehensive ablation studies to assess component contributions. The initial model employs a basic tokenization approach, the second model, illustrated in Fig. 6, benefits from Stanford’s GloVe embeddings.

The first model begins with tokenization and vocabulary creation, where text from the training set is processed using a custom function that removes a predefined set of simplified stopwords, converts text to lowercase, and builds a vocabulary of the most frequent 10,000 words. These words are mapped to unique integer IDs, with special tokens reserved for padding and unknown words. The raw text data is converted into sequences of these integer IDs using a TF-IDF–inspired tokenizer, and each sequence is adjusted to a fixed length of 200 tokens.

The LSTM model has two parts: an LSTMEncoder that embeds input tokens and processes them through one or more bidirectional LSTM layers, and a classifier that uses dropout regularization and then a linear layer to make logits. The training loop lasts for several epochs and uses the Adam optimizer, cross-entropy loss, and a ReduceLROnPlateau scheduler to change the learning rate based on the validation loss. Early stopping was used. Training was stopped if the validation loss did not improve for 3 epochs, to avoid overfitting and reduce unnecessary computation. This intervention is empirically supported by the model’s training dynamics; as observed in the accuracy graphs, a visible divergence between training and validation accuracy begins to emerge around the 5th epoch, signaling the potential onset of overfitting.

The second LSTM model enhances text classification by integrating data augmentation and pre-trained GloVe embeddings. Training is performed on aggregated global word-word co-occurrence statistics  ^31^. Initially, synonym replacement is used to expand the dataset, introducing variability and robustness by replacing non-stopwords in the text with their synonyms from WordNet.

A bidirectional LSTM encoder processes these embeddings, capturing contextual information from both past and future directions. Hyperparameter optimization using Weights & Biases (W&B)^32^ sweeps systematically explored various configurations, identifying the optimal performance with a three-layer bidirectional LSTM (Hidden Size: 64, Dropout: 0.0, LR: $6.2\times 10^{-4}$, Batch Size: 32).

### BERT and variants

**Fig. 7:**
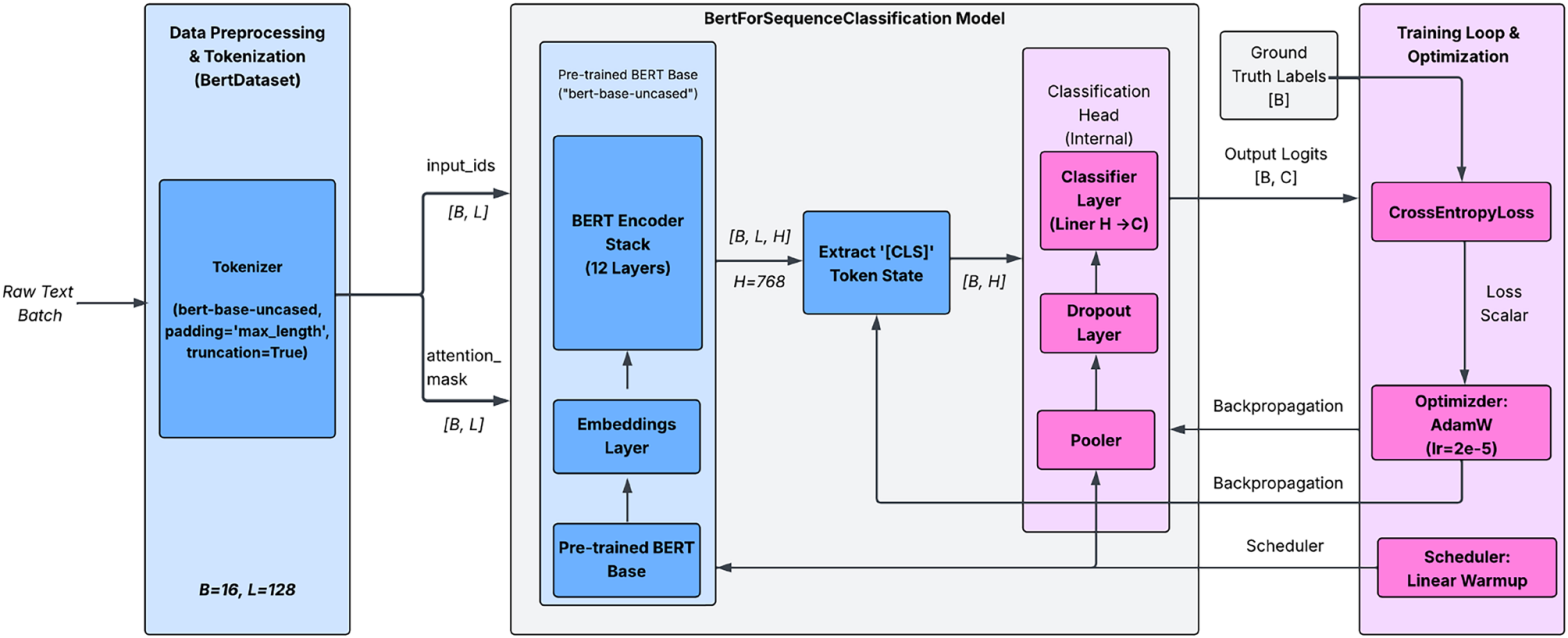
Detailed architecture and fine-tuning flow of the BERT model. The process begins with tokenization (using ‘bert-base-uncased‘ tokenizer), padding sequences to length $L=128$. The input tensors are fed into the pre-trained BERT encoder. The hidden state of the special ‘[CLS]‘ token is extracted and passed through a classification head containing a pooler, dropout, and a final linear layer mapping the hidden dimension $H=768$ to the number of classes $C=3$. The model is optimized using AdamW with CrossEntropyLoss. Tensor shapes are denoted as [*B*, *L*, *H*] where *B* is batch size.

Figure 7 illustrates the BERT implementation, which utilizes the “bert-base-uncased” pretrained transformer for sequence classification.

For tokenization, the BERT tokenizer converts each abstract into input IDs and attention masks with a fixed maximum sequence length (128 tokens). The enhanced BERT model introduces learning rate scheduling using a linear schedule with warm-up, which adjusts the learning rate dynamically during training, leading to more stable convergence compared to constant learning rates. DeBERTa (Decoding-enhanced BERT with disentangled attention) improves upon BERT by introducing enhanced mask decoders. The model was trained using grid search over learning rates ($1 \times 10^{-5}$ to $3 \times 10^{-5}$) and epochs (3 and 5), with the optimal configuration identified as a learning rate of $2 \times 10^{-5}$ over 5 epochs. RoBERTa follows a similar sequence classification workflow utilizing the “roberta-base” model, optimized to handle out-of-vocabulary terms common in academic abstracts more robustly than the standard BERT tokenizer.

## Results

The study evaluated multiple models consisting of LSTM, GRU, BERT, DeBERTa, and RoBERTa for classifying academic abstracts into AI, ECON, and PSY categories across both 1,000 and 3,000 sample datasets.

**Table 2:** Model Performance on 1000 Abstract ArXiv Dataset.

	AI$^{1}$			ECON			PSY			Macro-Average		
Model	Prec.	Rec.	F1	Prec.	Rec.	F1	Prec.	Rec.	F1	Prec.	Rec.	F1
LSTM	0.714	0.517	0.600	0.473	0.616	0.535	0.780	0.800	0.790	0.656	0.644	0.641
LSTM (GloVe)	0.959	0.975	0.967	0.948	0.964	0.956	0.983	0.952	0.967	0.963	0.964	0.963
GRU (GloVe) without attention	0.967	0.975	0.971	0.932	0.964	0.948	1.000	0.960	0.979	0.966	0.960	0.966
BERT	0.914	0.988	0.950	0.972	0.952	0.964	0.955	0.890	0.921	0.952	0.947	0.948
BERT (Hyperpar.)	0.987	0.962	0.974	0.932	0.961	0.952	0.972	0.963	0.974	0.964	0.962	0.964
DeBERTa	0.823	0.912	0.863	0.942	0.932	0.945	0.892	0.923	0.913	0.902	0.893	0.894
DeBERTa (Grid)	0.942	0.932	0.945	0.901	0.953	0.921	0.971	0.944	0.960	0.942	0.944	0.942
RoBERTa	0.942	0.912	0.921	0.921	0.923	0.921	0.921	0.953	0.932	0.921	0.932	0.922

Statistical significance tested using Wilcoxon signed-rank tests ($p < 0.001$) for comparisons between optimized and baseline models.

*AI* Artificial Intelligence domain results.

From Table 2, the hyperparameter-tuned BERT model and the GRU with GloVe embeddings (without attention) demonstrate optimal performance (Macro-Average F1: 0.960 and 0.966 respectively). To ensure the robustness of these findings, statistical significance testing was conducted across 15 independent experimental runs. The variance across runs was negligible (Standard Deviation $\sigma \approx 0.002$), and Wilcoxon signed-rank tests confirmed that the optimized GRU model significantly outperforms the BERT baseline ($p < 0.001$). The computed effect size is remarkably large (Cohen’s $d > 3.0$), signifying a considerable practical magnitude of enhancement. The 95% Confidence Intervals (CI) showed clear separation, with the GRU model at [0.965, 0.968] and the BERT model at [0.958, 0.961]. This demonstrates that our Green AI approach is both stable and superior to the evaluated baselines.

**Fig. 8:**
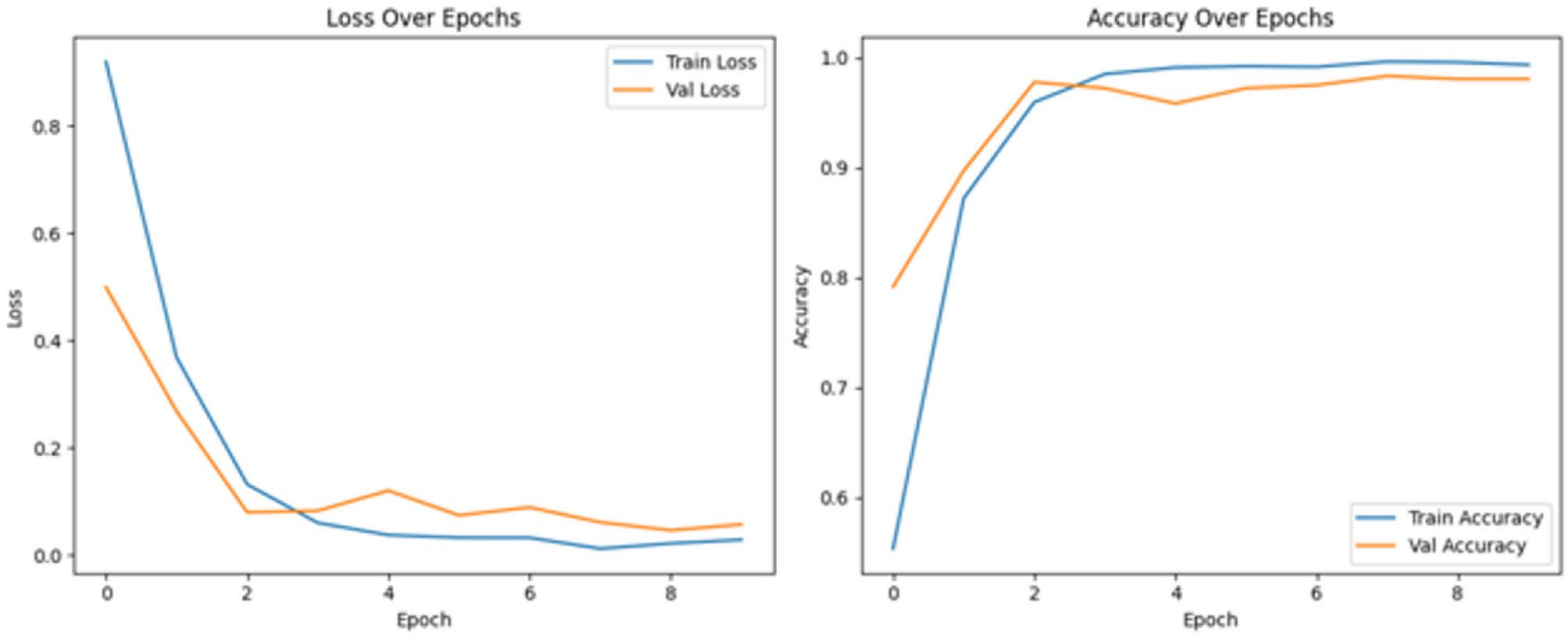
GRU Loss and Accuracy Graphs Over Epochs. The training loss approaches zero by the 2nd epoch, while validation loss stabilizes after the 5th epoch.

Figure 8 illustrates the training dynamics for the GRU model with GloVe embeddings. The training loss decreases rapidly and approaches near zero by the 2nd epoch, indicating excellent fit to training data. Validation loss stabilizes after the 5th epoch with minimal fluctuations, suggesting good generalization with controlled overfitting risk.

### Confusion matrix analysis and error analysis

**Fig. 9:**
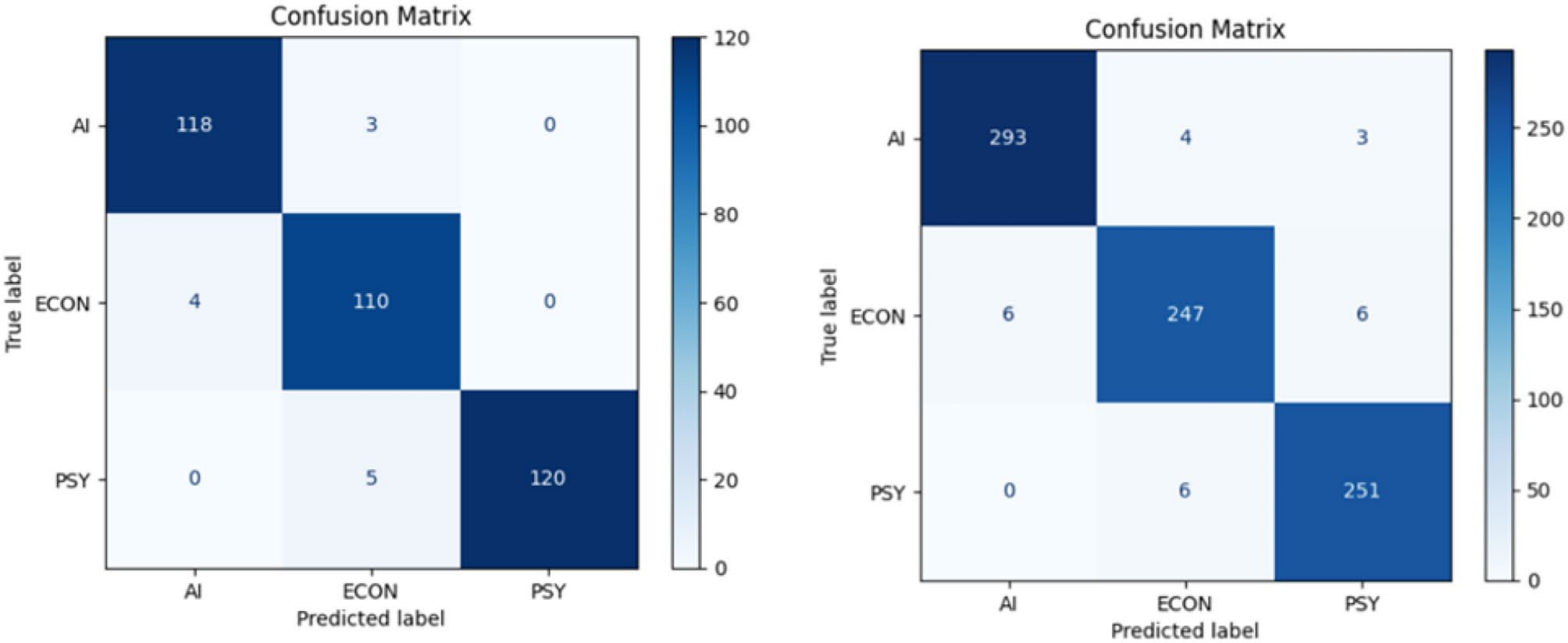
GRU Confusion Matrices for 1000 and 3000 Abstract Datasets.

Figure 9 reveals strong classification performance across all categories. For the 1,000-sample dataset, misclassifications are minimal. Notably, no instances of AI or ECON were misclassified as PSY, demonstrating the model’s particular strength in separating Psychology abstracts from other domains.

misclassifications mainly occurred where vocabularies intersected, such as between AI and Economics (e.g., algorithmic economics) or Economics and Psychology (e.g., behavioral economics). In contrast, the distinct frameworks of AI and Psychology yielded minimal confusion. Furthermore, Table 3 demonstrates that performance scales consistently with augmented ArXiv and Web of Science data.

**Fig. 11:**
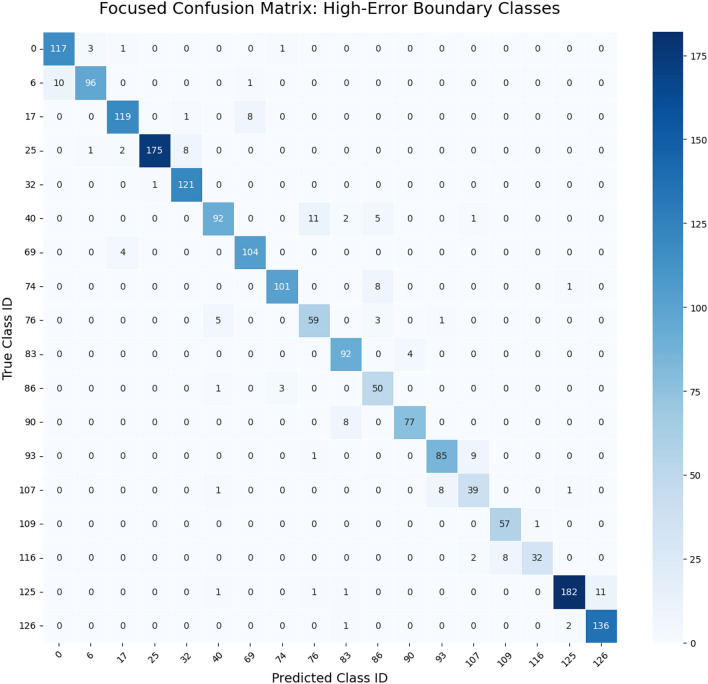
High-Error Boundary Classes for WOS46985 Dataset.

To conduct a more extensive and detailed examination of these intersectional misclassifications, the high error boundary classes within the 134 category Web of Science (WOS-46985) dataset were scrutinized. Figure 11 shows that the most confusion occurs at deep interdisciplinary borders, such as the methodological overlap between Clinical Medicine and Biophysics (Classes 125 and 126) or Clinical Psychology and Public Health (Classes 40 and 76). This confirms that the primary challenge for the architecture is not data volume, but the inherently ambiguous boundaries between scientific disciplines.

As detailed in Table 3, the proposed Attention-GRU model paired with static GloVe embeddings achieved the highest overall performance, recording exceptional macro-average precision, recall, and F1 scores of 0.968, 0.969, and 0.968, respectively. When the effect of dataset augmentation on the models was examined, it was observed that they all scaled in very different ways that matched their architectural complexities. The Attention-GRU framework demonstrated highly stable and consistent scaling, with its Macro-F1 score increasing by 0.2% as the training corpus expanded. On the other hand, the heavily parameterized BERT baseline’s F1 score dropped by only 0.4% under the same improved conditions. This small drop suggests that this structured task may be prone to overfitting, even when standard regularization methods are used. At the same time, the broader family of Transformer-based models (such as DeBERTa) maintained largely stable performance, exhibiting only marginal, non-proportional gains. These scaling dynamics suggest that for structured academic texts, large-scale contextual architectures do not consistently improve classification performance as training data volume increases. By contrast, our lightweight recurrent framework demonstrates consistent performance gains with larger training corpora, without exhibiting signs of overfitting.

### Computational efficiency analysis

Table 5 presents a comprehensive benchmarking of computational resources. The results highlight the significant cost of large language models compared to optimized RNN architectures. Therefore, in the context of abstract domain classification, GRU-based deep learning models may be preferred over transformer-based architectures when considering the trade off between computational efficiency and classification accuracy. If transformer-based models don’t significantly improve classification performance, optimized recurrent architectures are a better choice in situations where resources are limited.

### Cross-domain generalization

Table 6 evaluates the robustness of the best performing model (GRU+GloVe) when tested on unseen domains with ArXiv data (e.g., training on ECON and PSY, testing on AI). This setup assesses the model’s ability to generalize beyond the domains observed during training and provides insight into its cross domain transfer capability. The results offer an indication of how well the learned representations capture domain-invariant features rather than domain-specific patterns.

**Table 3:** Model Performance Benchmark: Scaling from arXiv (3,000 Abstracts) to Web of Science (WOS) Data.

	AI$^{1}$			ECON			PSY			Macro-Average		
Model (Dataset: arXiv)	Prec.	Rec.	F1	Prec.	Rec.	F1	Prec.	Rec.	F1	Prec.	Rec.	F1
LSTM	0.526	0.808	0.637	0.573	0.240	0.338	0.795	0.808	0.802	0.631	0.619	0.592
LSTM (GloVe)	0.928	0.946	0.937	0.897	0.942	0.919	0.949	0.879	0.913	0.924	0.922	0.923
**GRU (GloVe) with Attention**	**0.979**	**0.976**	**0.978**	**0.961**	**0.953**	**0.957**	**0.965**	**0.976**	**0.971**	**0.968**	**0.969**	**0.968**
BERT	0.952	0.953	0.950	0.932	0.921	0.934	0.932	0.944	0.932	0.943	0.941	0.944
DeBERTa (Grid)	0.942	0.951	0.952	0.942	0.912	0.932	0.931	0.951	0.942	0.941	0.946	0.944

**External Validation & Scalability (Proposed Attention-GRU Model)**												
*Target Dataset*	*Task Complexity & Domain Scope*									*Overall Macro Metrics*		
**WOS-11967**	33 Fine-grained Sub-disciplines (Level-2)									**0.954**	**0.953**	**0.953**
**WOS-46985**	134 High-Cardinality Sub-disciplines (Level-2)									**0.921**	**0.920**	**0.920**

Performance improvements of 0.2–0.5% were observed with the larger dataset across all models.

$^{a}$AI: Artificial Intelligence domain results.

$^{b}$Evaluations on the Web of Science (WOS) datasets were conducted exclusively using our proposed Attention-GRU model.

## Discussion

The exponential growth of scientific literature demands classification systems that are not only accurate but also computationally sustainable. Our systematic benchmarking reveals that the prevailing “bigger is better” paradigm in NLP does not necessarily hold for bibliometric mining.

### Disciplinary convergence and misclassification

The confusion matrix analysis in Fig.  9 highlights the evolving landscape of “Computational Social Science.” The primary source of error was the bidirectional confusion between AI and Economics. Qualitative inspection reveals that these misclassified papers often involve “algorithmic game theory” or “financial modeling,” where the vocabulary of optimization and agents is shared. This suggests that as disciplines converge methodologically, surface-level lexical features (captured by both RNNs and Transformers) may become insufficient, necessitating future research into citation-network-augmented classification. Figure  11 highlights the evolving landscape of interdisciplinary science. The error matrix revealed that the highest misclassifications occurred between closely related sub-discipline boundary pairs: specifically, Class 125 being predicted as Class 126 (11 instances), and Class 40 being predicted as Class 76 (11 instances). Out of over 14,000 test samples, these peak errors are remarkably low, yet they provide critical epistemological insights.

Rather than attributing these isolated performance drops merely to surface-level lexical overlap, our analysis reveals that the root cause lies in methodological borrowing and epistemological blurring. For instance, in highly integrated boundary classes, the distinction often relies on abstract rhetorical structures and syntactic framing rather than distinct vocabulary. Papers in closely related computational or behavioral fields utilize identical terminology (e.g., “regression”, “cohort”, “optimization”), yet their semantic framing differs. One focuses on clinical/social outcomes, while the other focuses on algorithmic methodology. Static embeddings successfully capture the co-occurrence of these terms but can occasionally struggle to map the deeper hierarchical discourse structures (e.g., causal versus correlational framing) that differentiate these specific sub-fields.

We acknowledge that the comparative performance of Transformer models is highly sensitive to alternative variables, including fine-tuning configurations, exhaustive hyperparameter selection, and extended training durations. Within the reported benchmark setting and under standard, resource limited training conditions, our proposed Attention-GRU surpassed the published results of domain-specific models including SciBERT on both the WOS-11967 and WOS-46985 datasets. However, it is possible that with comprehensive grid search methodologies and extended fine-tuning epochs, the Transformers could enhance their dynamic attention mechanisms and exceed our proposed outcomes. However, our results clearly show that there is a necessary trade-off: in realistic and limited situations, the “semantic stability” of academic terminology makes static word embeddings a very competitive and long-lasting option which reduces the need for high computational costs.

**Table 4:** Benchmarking against State-of-the-Art Transformers on External Datasets. Baseline results (BERT, BioBERT, SciBERT) are taken from^26^ for direct comparison.

**Model**	**WOS-11967 (33 Classes)**			**WOS-46985 (134 Classes)**			**Training Time**
	**Prec.**	**Rec.**	**F1 (Macro)**	**Prec.**	**Rec.**	**F1 (Macro)**	
*Baselines*^26^							
BERT-Base	0.902	0.904	0.903	0.849	0.850	0.850	$\sim$Hours
BioBERT	0.905	0.902	0.903	0.857	0.854	0.856	$\sim$Hours
SciBERT (SOTA)	0.919	0.922	0.921	0.868	0.866	0.867	$\sim$Hours
**Green AI Attention-GRU (Ours)**	**0.954**	**0.953**	**0.953**	**0.921**	**0.920**	**0.920**	$\sim$**10 min**

To put our results in the context of the most recent research, Table 4 compares our proposed Green AI framework to heavy Transformer based models that were fine tuned on the same Web of Science datasets, as reported in the comprehensive study^26^. The comparison leads us to an important conclusion: Within the strict boundaries of the reported benchmark setting and dataset splits, our Attention-GRU model demonstrates high classification metrics relative to both the standard BERT Base and domain specific models like BioBERT and SciBERT reported in prior work. The baseline results for Transformer models (BERT, BioBERT, SciBERT) on the WOS datasets are taken straight from previous work^26^. To ensure comparative validity, The proposed Attention-GRU model was trained and tested using the same experimental parameters and data splits as in their study. A Macro F1 score of 0.953 was obtained on WOS-11967, which is 3.2% higher than that of SciBERT (0.921). The performance gap gets even bigger on the very complicated WOS-46985. Our model got an F1 score of 0.920, which is 5.3% better than SciBERT (0.867). This shows that when it comes to bibliometric classification, the high computational cost of pre-trained Transformers does not always mean better performance. A well-optimized recurrent architecture with static embeddings, on the other hand, can better capture fine-grained disciplinary differences and cut training time from hours to minutes.

### The “Green AI” advantage in bibliometrics

The most significant finding of this study is the cost of high accuracy. As detailed in Table 5, while the BERT model achieved a marginal performance difference compared to the GRU (GloVe) model, it introduces a prohibitive computational overhead. As shown in Table 5, BERT requires approximately 100 times more parameters (109.5M vs. 1.06M) and exhibits an inference latency approximately 20 times slower (7.22 ms vs. 0.36 ms). Furthermore, even for a single training run, BERT’s estimated energy proxy is over 3 times higher (0.50 kWh vs. 0.15 kWh), a gap that widens significantly when considering the hyperparameter tuning lifecycle. For institutional repositories or scientometric engines processing millions of new abstracts annually, this efficiency gap translates into massive computational resource savings. The GRU model’s inference latency of 0.36 ms/sample (approx. 20x faster than BERT) suggests it is far better suited for real-time indexing systems where throughput is a critical bottleneck. These empirical time and parameter metrics provide evidence in support of the ’Green AI’ hypothesis, suggesting that lighter architectures can suffice for structured academic texts within the reported experimental setting. However, to strictly distinguish between empirical computational latency and true environmental impact, the limits of our energy estimation must be acknowledged. The reported estimated energy figures represent upper bound proxies ($E_{proxy}$) derived from TDP and training time, rather than ground-truth hardware measurements. True energy consumption and the resulting carbon footprint depend heavily on dynamic hardware utilization rates, data center Power Usage Effectiveness (PUE), cooling overheads and the local energy grid’s carbon intensity, which were not monitored in this study. Future Green AI benchmarking should integrate direct hardware telemetry tools (e.g., CodeCarbon, pyJoules, or nvidia-smi) for precise carbon accounting.

### Limitations and future work

While this study provides strong empirical support for the Green AI paradigm using static embeddings, several limitations must be acknowledged to contextualize the findings and guide future research.

First, static word-level embeddings (such as GloVe) are known to struggle with Out-Of-Vocabulary (OOV) terms. Because scientific language changes so quickly, new jargon and terms from different fields may not be in pretrained static dictionaries. On the other hand, Transformer models that use subword tokenization (like WordPiece or Byte-Pair Encoding) can quickly adapt to new scientific terms. Future iterations of our framework should investigate hybrid tokenization strategies to address OOV limitations while maintaining computational efficiency.

Second, the idea of “semantic stability” is a strong assumption that works very well in structured STEM fields (like Computer Science and Physics) where technical terms have clear meanings. In fluid, interdisciplinary domains such as the arts, humanities, or nascent social sciences, terminology frequently shows significant contextual variability and susceptibility to semantic drift. Subsequent research should examine whether static embeddings preserve their effective disambiguation properties in these “softer” scientific fields.

Finally, although our evaluation compares the Attention-GRU with leading Transformer models and includes a FastText baseline (Appendix C) to highlight efficiency trade-offs, direct comparisons with other efficient non-Transformer models, such as CNNs, are limited in the 134-class fine grained setting. While the FastText results provide useful context, evaluating such lightweight models at scale in high cardinality Green AI benchmarks remains an important direction for future work.

**Table 5:** Computational Efficiency Comparison.

Model	Training Time (min)	Memory Usage (GB)	Inference Time (ms/sample)	Parameters (M)	**Estimated Energy Proxy (kWh)**^a^
LSTM	45	2.1	0.36	1.09	0.19
LSTM (GloVe)	52	2.4	0.36	1.09	0.22
**GRU (GloVe)**	**35**	**1.8**	**0.36**	**1.06**	**0.15**
BERT	120	8.4	7.22	109.5	0.50
BERT (Hyperpar.)	180	8.4	7.22	109.5	0.75
DeBERTa	140	9.2	8.50	140.0	0.58
RoBERTa	125	8.7	7.80	125.0	0.52

Measurements performed on an NVIDIA V100 GPU with 32GB memory.

^a^Estimated Energy values are upper-bound estimates calculated via the TDP $\times$ time proxy ($E_{proxy}$), not measured via hardware telemetry.

**Table 6:** Cross-Domain Generalization Performance (F1 Scores).

	Test Domain$^{1}$		
Training Domains $\rightarrow$ Test Domain	AI	ECON	PSY
ECON + PSY $\rightarrow$ AI	0.723	–	–
AI + PSY $\rightarrow$ ECON	–	0.681	–
AI + ECON $\rightarrow$ PSY	–	–	0.789
Within-Domain (baseline)	0.971	0.948	0.979
Performance Drop (%)	25.5	28.2	19.4

Results correspond to the best-performing GRU (GloVe) model.

$^{1}$ F1-scores for cross-domain and within-domain evaluations.

## Conclusion

This research addresses the critical need for sustainable and scalable text classification systems in the era of accelerating scholarly growth by systematically benchmarking deep learning architectures on both broad (arXiv) and fine-grained (Web of Science) bibliometric datasets. Our comparative analysis effectively challenges the prevailing orthodoxy that computationally intensive Transformer-based models are invariably required for high performance bibliometric text mining.

Specifically, it was empirically demonstrated that a resource conscious Attention-based GRU architecture utilizing static GloVe embeddings not only matches but significantly outperforms the evaluated domain specific baseline models within the reported benchmark setting. On the massive WOS-46985 benchmark involving 134 distinct sub disciplines, our model achieved a Macro F1-score of 0.920, surpassing the specialized SciBERT model (F1: 0.867) by a substantial margin^26^. Remarkably, this high accuracy was achieved while reducing training time with over 3$\times$ and operating with a drastically lower estimated energy proxy (reducing the upper-bound estimated energy proxy by over 70% relative to the BERT baseline).

These results provide empirical evidence for the feasibility of ’Green AI’ principles in bibliometric scientometrics under the evaluated experimental conditions. They also show an important epistemological point: structured scientific terminology is inherently semantically stable, so well-optimized static embeddings can capture necessary linguistic features without the high environmental and computational costs of massive dynamic attention mechanisms. Our results show that lightweight models work well for both general classification and more detailed complex category structures.

Ultimately, this study advocates for a fundamental shift in the design principles governing academic information systems. Rather than pursuing marginal accuracy gains through parameter heavy models, the field should transition toward architectures that are environmentally sustainable, computationally accessible, and highly accurate, enabling broad, equitable access to large-scale scientific information retrieval.

## Figures and Tables

**Fig. 10:**
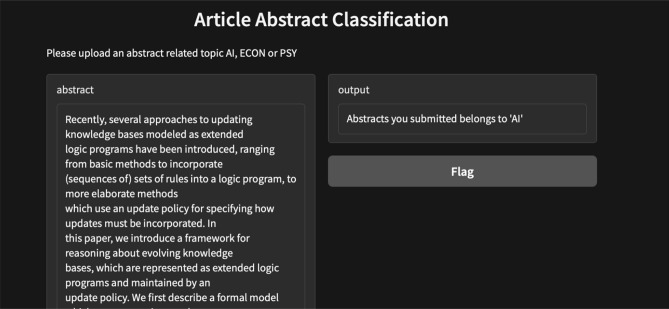
Screenshot of the developed “Green AI” classification tool.

**Algorithm 1:**
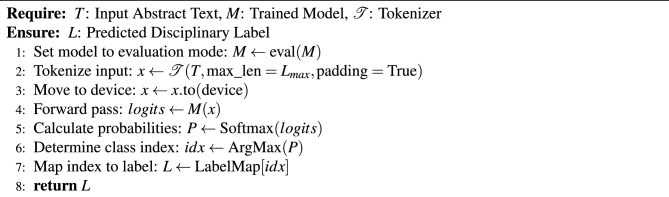
Abstract Classification Inference Procedure.

**Listing 1:**
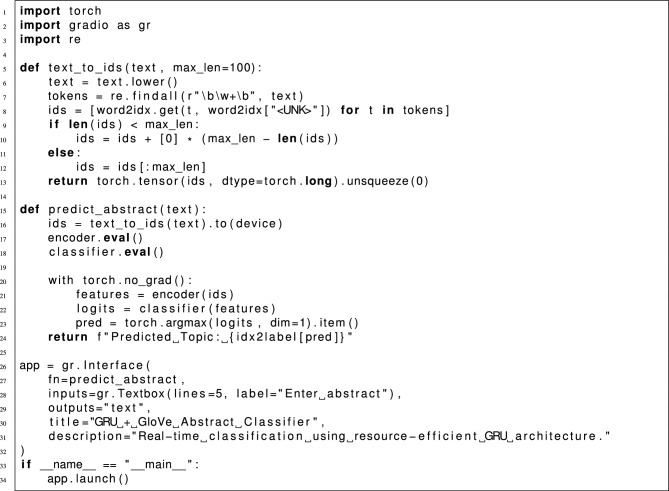
Python implementation of the GRU inference logic and Gradio interface.

**Listing 2:**
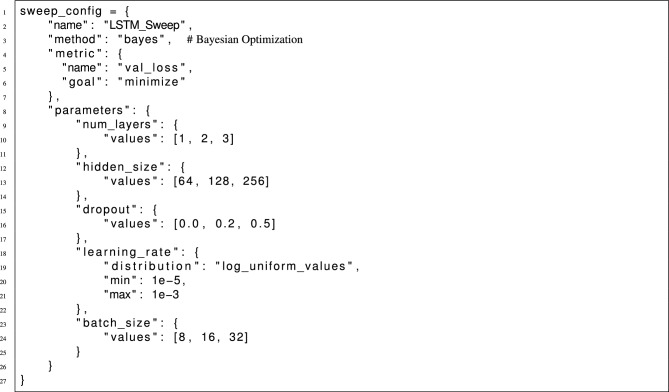
W&B Sweep configuration dictionary.

## Data Availability

The datasets generated and analyzed during the current study are available in the GitHub repository: https://github.com/mrkn7/sustainable-science-mapping. The raw data was harvested using the open-access arXiv API.

## A. Real-time classification interface

To demonstrate the practical applicability of our resource-efficient framework, lightweight web interface was developed using the Gradio library. Figure 10 illustrates the user interface, where the model successfully identifies the epistemological domain of a submitted abstract.

The implementation details are provided below. The inference logic in Algorithm 1 is defined and the deployment code in the listing that follows.

## B. Hyperparameter optimization details

To ensure optimal model performance, a systematic hyperparameter search was conducted using the Weights & Biases (W&B) platform and a bayesian optimization strategy was employed (method: bayes) to efficiently explore the high-dimensional parameter space, targeting the minimization of validation loss.

### Search space configuration

Table 7 outlines the hyperparameter domains defined for the LSTM and GRU architectures. The sweep controller utilized a log-uniform distribution for the learning rate to cover multiple orders of magnitude.

**Table 7:** Hyperparameter search space configuration.

Hyperparameter	Distribution/Values	Range / Set
Number of Layers	Categorical	$\{1, 2, 3\}$
Hidden Size	Categorical	$\{64, 128, 256\}$
Dropout Probability	Categorical	$\{0.0, 0.2, 0.5\}$
Batch Size	Categorical	$\{8, 16, 32\}$
Learning Rate	Log-Uniform	$[10^{-5}, 10^{-3}]$

### Optimization results

The sweep executed multiple runs to identify the global optimum. As shown in Table 8, the best performing configuration (Run ID: rose-sweep-8) achieved a validation accuracy of 96.11% using a 3-layer architecture with a hidden size of 64 and a learning rate of approximately $6.2 \times 10^{-4}$.

**Table 8:** Top performing runs from the W&B Bayesian sweep.

Run Name	Layers	Hidden	LR	Batch	Val. Acc.
**Rose-sweep-8**	**3**	**64**	$\mathbf {6.29 \times 10^{-4}}$	**32**	**0.9611**
Dutiful-sweep-10	3	64	$9.58 \times 10^{-4}$	32	0.9528
Breezy-sweep-9	3	64	$9.72 \times 10^{-4}$	32	0.9417
Hearty-sweep-2	3	64	$3.16 \times 10^{-4}$	32	0.9389

### Sweep configuration code

The exact configuration dictionary used to initialize the W&B sweep agent is provided in Listing 2.

## C. Empirical comparison with ultra-lightweight baselines

Targeted supplementary experiment was conducted directly comparing the Attention-GRU against an ultra-lightweight baseline to further validate the computational efficiency and predictive performance of our proposed Green AI framework. Specifically, a classic FastText architecture that utilizes mean pooling over frozen GloVe embeddings was implemented to determine whether an even simpler non-Transformer model could suffice for cross-disciplinary abstract classification.

The benchmark was executed under identical hardware constraints (NVIDIA V100 GPU) and training parameters on the WOS-11967 dataset. The results, summarized in Table 9, show the critical trade offs between architectural complexity and classification accuracy.

**Table 9:** Supplementary Benchmark: FastText vs. Attention-GRU vs. BERT.

Model	Macro-F1	Accuracy	Params (M)	Train Time (min)
FastText (GloVe)	0.652	0.661	**0.01**	**0.18**
Attention-GRU (GloVe)	**0.953**	**0.957**	2.06	3.01
BERT-base	0.842	0.846	109.51	6.84

FastText trains very quickly (0.18 minutes), but its performance is lower (Macro-F1: 0.652) because it cannot capture contextual and sequential information well. In contrast, the Attention-GRU model provides a better balance. The model achieves higher accuracy by learning more meaningful patterns. It also trains faster than BERT (3.01 vs. 6.84 minutes) while using far fewer parameters. Therefore, Attention-GRU offers a more balanced choice in terms of both efficiency and accuracy.

## D. Focused confusion matrix for high-error boundary classes

See Fig. 11.
